# Dynamics of Mono- and Dual-Species Biofilm Formation and Interactions Between *Paracoccidioides brasiliensis* and *Candida albicans*

**DOI:** 10.3389/fmicb.2020.551256

**Published:** 2020-10-14

**Authors:** Lariane Teodoro Oliveira, Kaila Petronila Medina-Alarcón, Junya de Lacorte Singulani, Nathália Ferreira Fregonezi, Regina Helena Pires, Rodrigo Alex Arthur, Ana Marisa Fusco-Almeida, Maria José Soares Mendes Giannini

**Affiliations:** ^1^Department of Clinical Analysis, School of Pharmaceutical Sciences, São Paulo State University-UNESP, Araraquara, Brazil; ^2^Laboratory of Mycology and Environmental Diagnosis, University of Franca, Franca, Brazil; ^3^Department of Preventive and Community Dentistry, Dental School, Federal University of Rio Grande do Sul, Porto Alegre, Brazil

**Keywords:** *Candida albicans*, *Paracoccidioides brasiliensis*, dual-species biofilm, oral cavity, *Galleria mellonella*

## Abstract

The oral cavity is a highly diverse microbial environment in which microorganisms interact with each other, growing as biofilms on biotic and abiotic surfaces. Understanding the interaction among oral microbiota counterparts is pivotal for clarifying the pathogenesis of oral diseases. *Candida* spp. is one of the most abundant fungi in the oral mycobiome with the ability to cause severe soft tissue lesions under certain conditions. *Paracoccidioides* spp., the causative agent of paracoccidioidomycosis, may also colonize the oral cavity leading to soft tissue damage. It was hypothesized that both fungi can interact with each other, increasing the growth of the biofilm and its virulence, which in turn can lead to a more aggressive infectivity. Therefore, this study aimed to evaluate the dynamics of mono- and dual-species biofilm growth of *Paracoccidioides brasiliensis* and *Candida albicans* and their infectivity using the *Galleria mellonella* model. Biomass and fungi metabolic activity were determined by the crystal violet and the tetrazolium salt reduction tests (XTT), respectively, and the colony-forming unit (CFU) was obtained by plating. Biofilm structure was characterized by both scanning electronic- and confocal laser scanning- microscopy techniques. Survival analysis of *G. mellonella* was evaluated to assess infectivity. Our results showed that dual-species biofilm with *P. brasiliensis* plus *C. albicans* presented a higher biomass, higher metabolic activity and CFU than their mono-species biofilms. Furthermore, *G. mellonella* larvae infected with *P. brasiliensis* plus *C. albicans* presented a decrease in the survival rate compared to those infected with *P. brasiliensis* or *C. albicans*, mainly in the form of biofilms. Our data indicate that *P. brasiliensis* and *C. albicans* co-existence is likely to occur on oral mucosal biofilms, as per *in vitro* and *in vivo* analysis. These data further widen the knowledge associated with the dynamics of fungal biofilm growth that can potentially lead to the discovery of new therapeutic strategies for these infections.

## Introduction

An important step in the development of infectious diseases involves the ability of microorganisms to adhere to host surfaces. Adherence is a widely distributed biological phenomenon and is also the first step in the process of biofilm formation (Verstrepen and Klis, 2006; Pitangui et al., 2012; Martinez and Casadevall, 2015). Biofilm arrangement can protect fungal pathogens from host defenses and reduce the diffusion of antifungal drugs (Martinez and Casadevall, 2006; Kernien et al., 2017; Tulasidas et al., 2018; Thomaz et al., 2020). Clinically, fungal biofilms can adhere to both abiotic surfaces, comprised of medical and dental devices, as well as to mucous membranes (Kernien et al., 2017). Many clinically relevant fungi can grow as biofilms, including *Candida* spp. (Hawser and Douglas, 1994) and *Paracoccidioides* spp. (Sardi Jde et al., 2015; Cattana et al., 2017).

Paracoccidioidomycosis (PCM) is a human systemic mycosis which assumes increasing clinical importance due to the increase in their frequency and mortality rates in South and Central America (Coutinho et al., 2002). *Paracoccidioides* spp. are dimorphic fungi that change their morphology according to the environmental temperature, with a transition from mold at 25°C to yeast at 37°C in human lungs (San-Blas and Vernet, 1977; Gauthier, 2017). Although the primary route of PCM infection is pulmonary, the disease is frequently diagnosed by extensive, ulcerative and painful buccal manifestations corresponding to the clinical form of the multifocal type (Franco et al., 1987; Mendes et al., 2017; de Arruda et al., 2018).

*Candida* spp. is a commensal fungus found in the gastrointestinal tract, on oropharyngeal and vaginal mucosa of humans, with the ability to cause mucosal infections under certain conditions, such as immunosuppression, radiotherapy, antibiotics, and corticosteroids (Fotos et al., 1992; Akpan and Morgan, 2002; Epstein et al., 2002; Fangtham et al., 2014). *Candida albicans* is one of the most abundant fungi in the oral mycobiome (Ramage et al., 2004; Dongari-Bagtzoglou et al., 2009; Millsop and Fazel, 2016). Increasing evidence indicate that the coexistence of *C. albicans* and oral bacteria as well as other interactions with different kingdoms affect both the dynamics of biofilm growth and the course and severity of mucosal lesions, as reviewed by Negrini et al. (2019).

Considering that both *Candida* and *Paracoccidioides* may co-exist in the oral cavity, the impact of such fungal-fungal interaction on biofilm formation and its pathogenic potential are still unknown. Therefore, this study aimed to evaluate the dynamics of mono- and dual-species biofilm formed by *P*. *brasiliensis* plus *C. albicans* and their infectivity using the *Galleria mellonella* model. Our hypothesis is that both fungi interact with each other boosting the biofilm growth, that in turn could lead to a more aggressive infectivity.

## Materials and Methods

### Fungal Strains and Growth Conditions

*Paracoccidioides* strains are currently classified as a complex of five cryptic species (*P. brasiliensis, P. americana, P. restrepiensis, P. venezuelensis*, and *P. lutzii*) distinguished by the phylogeny findings and studies using molecular techniques (Matute et al., 2006; Carrero et al., 2008; Turissini et al., 2017; De Macedo et al., 2019). We used the *P. brasiliensis* strain (former S1 phylogenetic group, Pb 18 strain, De Macedo et al., 2019), originally isolated from a case of pulmonary paracoccidioidomycosis in São Paulo, SP, Brazil. This strain belongs to the collection of the Clinical Mycology Laboratory, Department of Clinical Analysis, School of Pharmaceutical Sciences, UNESP. *C. albicans* ATCC 90028 was also used and it was cultured on Sabouraud dextrose agar (Difco—BD Biosciences, Sparks, MD, United States) and incubated at 37°C for 48 h. *P. brasiliensis* (Pb18) was cultured in Fava-Netto standard medium (Fava Netto et al., 1969) and incubated at 37°C for 5 days.

### *In vitro* Biofilm Growth

The inocula were prepared as previously published, as per the *C. albicans* biofilm formation protocol (Jin et al., 2004). Briefly, a suspension of each fungus was standardized in Brain Heart Infusion (BHI) broth at 1 × 10^6^ cells/mL using a hemocytometer. In all experiments, the fungal cell viability was evaluated using trypan blue (do Carmo Silva et al., 2015). Monospecies biofilms were developed on a polystyrene 96-well microtiter plate by adding 200 μL of *C. albicans* or *P. brasiliensis* standard inoculum to six wells. The plates were incubated at 37°C for 168 h with both fungi. The medium was changed during the total incubation time every 24 h. To compare the growth rates, both the biofilms were incubated for 168 h according to the kinetics for the biofilm of *P. brasiliensis* previously determined by our group (Sardi Jde et al., 2015).

Dual-species biofilms were developed on a 96-well microtiter plate by adding 100 μL of *P. brasiliensis* and 100 μL of *C. albicans* standard inoculum to six wells and by incubating them for 168 h at 37°C. Based on the assumption that *C. albicans* already exists in the oral microenvironment, we developed a second group of dual-species biofilms. In this group, we inoculated 100 μL of *P. brasiliensis* standard inoculum to a preformed 12 h *C. albicans* biofilm at 37°C. This was followed by incubation for a total period of 168 h at 37°C. In both dual-species biofilm groups, the medium was changed every 24 h of incubation.

### Quantitative Biofilm Analysis

#### Biomass Quantification

Biofilm biomass was quantified based on the crystal violet (CV) assay (Peeters et al., 2008) with modifications. One microplate containing mono-species and dual-species biofilms was subjected to the CV methodology every 24 h until a 168 h total period. After each 24 h of incubation, the plates were washed with PBS to remove non-adherent cells. The biofilms were fixed with 100 μL of 99% methanol (Sigma-Aldrich, São Paulo, Brazil). After 15 min, the supernatants were removed, and the plates were air-dried. Then, 100 μL of 0.1% CV solution was added to all wells. After 20 min, the excess CV was removed by washing with PBS. Finally, the bound CV was released by adding 150 μL of 33% acetic acid (Sigma-Aldrich, São Paulo, Brazil). The absorbance was measured at 570 nm using a microplate reader (Epoch, Biotek). All steps were carried out at room temperature. Three independent experiments were performed with six replicates each.

#### Biofilm Metabolic Activity Assessment

The biofilm metabolic activity was assessed through the XTT (2.3-bis (2-methoxy-4-nitro-5-sulfophenyl)-5-[carbonyl (phenylamino)]-2H-tetrazolium hydroxide) reduction assay. About 50 μL of XTT salt solution (1 mg/mL in PBS) and 4 μL of menadione solution (1 mM in ethanol; Sigma-Aldrich, São Paulo, SP., Brazil) were added to each well of the 96-well plate and incubated at 37°C for 3 h. The test was carried out every 24 h until a total period of 168 h for both mono-species and dual-species biofilms. The absorbance was measured using a microplate reader (Epoch, Biotek) at 490 nm (Martinez and Casadevall, 2006; Silva et al., 2010). In all assays, the culture media were included as negative control. Three independent experiments were performed with six replicates each.

#### Counts of Fungal Viable Cells

After biofilm formation, at different point times (0, 24, 48, 72, 96, 120, 144, and 168 h), the wells were washed thrice with PBS to remove loosely adhered cells. The biofilms were then mechanically disrupted, filling the wells with 100 μL of BHI broth and then vigorously mixed. Each individual well content was transferred to microtubes containing 900 μL of BHI broth. The suspensions were serially diluted and plated onto BHI agar supplemented with 1% glucose, 5% of *P. brasiliensis* 339 culture filtrate and 4% fetal bovine serum and caspofungin 0.25 μg/mL (for *P. brasiliensis* counts) and on Sabouraud dextrose agar (for *C. albicans* counts) that were incubated at 37°C for 10 days and 48 h, respectively. The number of viable fungal colony-forming units (CFU) was determined under a stereomicroscope and the results were expressed as log_10_ CFU/mL. Three independent experiments were performed with six replicates each.

### Structural Biofilm Analysis

#### Confocal Laser Scanning Microscopy (CLSM)

The standardized *P. brasiliensis* inoculum was treated with carboxyfluorescein diacetate succinimidyl ester dye (CFSE, 100 μM/mL, BioChemika), kindly provided by Dr Paula Aboud Barbugli from Araraquara School of Dentistry (São Paulo State University - UNESP) for 30 min at 37°C. The fungi cells were washed with PBS and resuspended in BHI medium by restoring the initial volume. This inoculum was then incubated with 12 h preformed *C. albicans* biofilms in 24-well plates. Mono-species biofilms of *P. brasiliensis* or *C. albicans* grown for a time that allowed their maturation (144 and 48 h, respectively) were used as controls (Sardi Jde et al., 2015). For the formation of dual-species biofilms of *P. brasiliensis* and *C. albicans*, equitable volumes of the standardized inoculum of the two organisms were co-cultivated and grown for 120 h, the time of greatest metabolic activity determined by the XTT test (section “Biofilm Metabolic Activity Assessment”). Next, the biofilms were washed carefully with sterile PBS to remove non-adherent cells. The biofilms were stained with calcofluor white (1 g/L; Sigma-Aldrich St. Louis, MO, United States) for 30 min at room temperature and washed again with PBS. The biofilms were photographed under a 20× dry immersion objective (numerical aperture 1.0) using a Zeiss LSM 800 confocal microscope. Each field of view was photographed using a 405 nm laser to capture calcofluor with an emission range up to 450 and 488 nm for CFSE detection with an emission range up to 540 nm.

#### Scanning Electron Microscopy (SEM)

To access the morphology, the biofilms were formed in 24-well plates according to previously published protocols (Morris et al., 1997; Ells and Truelstrup Hansen, 2006) with modifications. Mono-species biofilms of *P. brasiliensis* or *C. albicans* grown at 37 C for 144 and 48 h, respectively, were used as controls. Dual-species biofilms were formed over a period of 120 h. Next, the plates were washed with PBS to remove non-adherent cells. The biofilms were fixed with 1,000 μL of 2.5% glutaraldehyde solution (Sigma-Aldrich, St Louis, MO, United States) in sterile distilled water overnight (for both *P. brasiliensis* mono-species and dual-species biofilms) or for 3 h (*C. albicans* mono-species biofilms), washed thrice with PBS and sequentially dehydrated with ethanol (50–100%) at room temperature. All samples were dried in a 780A Samdri desiccator (Rockville, MD, United States). Subsequently, the samples were mounted on aluminum and silver cylinders and disposed in a high vacuum evaporator (Denton Vacuum Desk V, Jeol, Moorestown, NJ, United States) for gold coating. The topographic images of biofilms were captured under a scanning electron microscope (Jeol JSM- 6610LV, Peabody, MA, United States).

#### Survival Assay Using *G. mellonella* Model

Survival assay was performed according to the previous protocol (Scorzoni et al., 2015), with some modifications. *G*. *mellonella* larvae (School of Pharmaceutical Sciences, São Paulo State University–UNESP) with a body weight ranging from 150 to 200 mg were randomly chosen for the experiments. The larvae were separated into six infection groups, each group consisting of 9–10 larvae which were kept in Petri plates at 37°C overnight, prior to use. To obtain planktonic cells, *C. albicans* was grown in Sabouraud agar at 25°C for 48 h and *P. brasiliensis* was grown in BHI broth supplemented with 1% glucose at 37°C and 150 rpm for 72 h. Pre-formed biofilms of *C. albicans* for 12 h and *P. brasiliensis* for 120 h in a 24-well plate were scrapped using a sterile tip and utilized for inoculum preparation. The inoculum of fungi for planktonic cells or cells from biofilms were prepared in PBS and counted using a hemocytometer. For each group, the larvae were injected at a concentration of 1 × 10^6^ cells/larvae of *C. albicans* or *P. brasiliensis* (2 × 10^6^ cells/larvae total for co-infection) into the last pro-leg using a 10 μL Hamilton syringe (Chibebe Junior et al., 2013; Scorzoni et al., 2015; de Barros et al., 2018). Survival larvae were monitored daily for up to 7 days of infection. Larvae were considered dead when they displayed no movement in response to touch. Larvae inoculated once or twice with sterile PBS were used as controls. Three independent experiments were performed, resulting in an *n* = 27–30 larvae/group.

### Statistical Analysis

The statistical analysis was conducted using the GraphPad Prism 5.0 software (GraphPad Software, Inc., San Diego, CA). The results were presented as mean ± standard deviation and compared by analysis of variance (ANOVA) followed by Bonferroni test. The data from the survival of *G. mellonella* larvae were plotted as Kaplan–Meier survival curves and compared using log-rank tests. Statistical significance was considered when *p* < 0.05.

## Results

### Biomass Quantification

**FIGURE 1:**
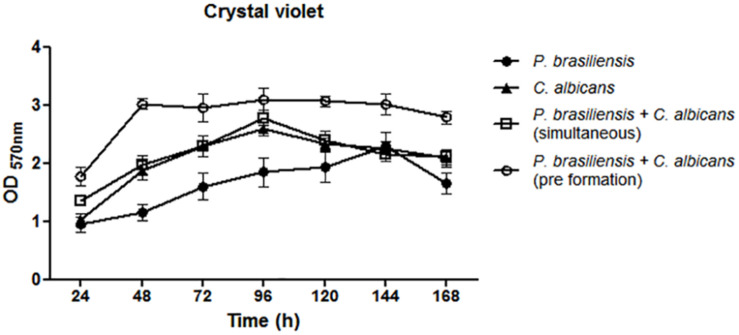
Quantification of mono and dual-species biofilm biomass by crystal violet methodology. Co-cultivated dual-species biofilms and dual-species biofilms formed by the addition of *P. brasiliensis* inoculum to preformed 12 h *C. albicans* biofilms. Three independent experiments with six replicates each were performed. Error bars indicate the standard error. Statistical significances were calculated by using analysis of variance (ANOVA) followed by Bonferroni test.

Overall, a biomass increase of all tested biofilms was observed in the period from 24 to 96 h growth, and this increase extended to up to 144 h in the case of *P. brasiliensis* mono-species biofilm (Figure 1). Comparison of mono-species with dual-species biofilms was performed using the analysis of variance (ANOVA) followed by Bonferroni test. Two different approaches have been done in relation to the dual-species genera interaction: (1) *P. brasiliensis* and *C. albicans* incubated simultaneously; (2) *P. brasiliensis* incubated after *C. albicans* pre-formed biofilm. The biomass of dual-species biofilms formed after *P. brasiliensis* was added to pre-formed 12 h *C. albicans* biofilms was statistically higher than the biomass of simultaneously formed dual-species biofilms at 48 h growth (*p* < 0.05). The biomass of *P. brasiliensis* mono-species biofilms was statistically lower than the biomass of simultaneously formed dual-species biofilms at 48 h (*p* < 0.01), 72 h (*p* < 0.05), and 96 h (*p* < 0.001) of growth and lower than the biomass of dual-species biofilms grown from *P. brasiliensis* added to 12 h pre-formed *C. albicans* biofilms at 48, 72, and 96 h (*p* < 0.001) and at 120 and 168 h (*p* < 0.01) of growth. No difference was found in the biomass of *C. albicans* as a mono-species biofilm compared to simultaneously formed dual-species biofilms in all periods analyzed, except the biomass at 48 h that was statistically lower than the biomass of dual-species biofilms grown from *P. brasiliensis* added to 12 h preformed *C. albicans* biofilms (*p* < 0.01).

### Biofilm Metabolic Activity

**FIGURE 2:**
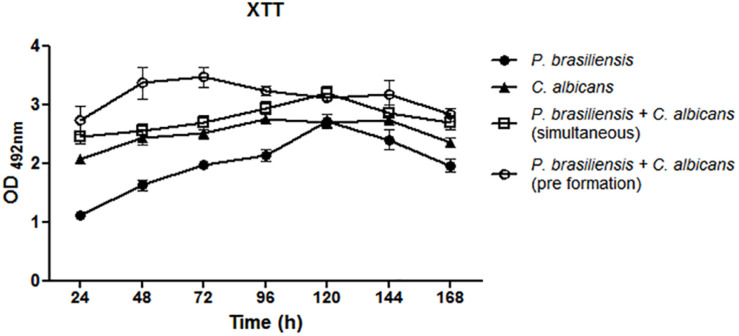
Metabolic activity of mono- and dual-species biofilms. Three independent experiments with six replicates each were performed. Co-cultivated dual-species biofilms and dual-species biofilms formed by the addition of *P. brasiliensis* inoculum to preformed 12 h *C. albicans* biofilms. Error bars indicate the standard error. Statistical significances were calculated by using analysis of variance (ANOVA) followed by Bonferroni test.

*P. brasiliensis* biofilm presents an increase in the metabolic activity from 24 h to up to 120 h of growth, whereas the *C. albicans* biofilm exhibits an increase in metabolic activity up to 48 h. Dual-species biofilms showed higher metabolic activity, mainly when *P. brasiliensis* was added to preformed 12 h *C. albicans* biofilms and there was a slight reduction after 72 h. Comparison of mono-species biofilms with dual-species was performed using analysis of variance (ANOVA) followed by Bonferroni test. The metabolic activity of *P. brasiliensis* biofilms was statistically lower than simultaneously formed dual-species biofilms at all time points (*p* < 0.001 for 24, 48, 72, 96, and 168 h and *p* < 0.05 for 120 and 144 h) and statistically lower than dual-species biofilms grown from *P. brasiliensis* added to preformed 12 h *C. albicans* biofilms at all time points (*p* < 0.001) except at 120 h of growth. Metabolic activities of *P. brasiliensis* and *C. albicans* biofilms were statistically lower than simultaneously formed dual-species biofilms only at 120 h of growth (*p* < 0.01) and at 24 h (*p* < 0.05), 48 and 72 h (*p* < 0.001) in comparison to dual-species biofilms when *P. brasiliensis* was added to preformed 12 h *C. albicans* biofilms. The metabolic activity of simultaneously formed dual-species biofilms was statistically lower than those in other conditions at 48 and 72 h of growth (*p* < 0.01) (Figure 2).

### Determination of Viable Cells as CFU/mL

**FIGURE 3:**
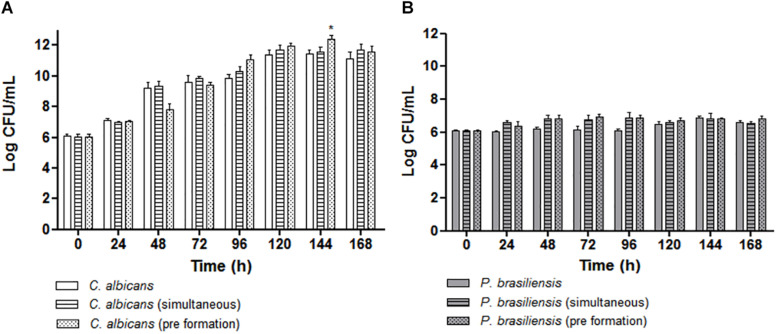
Quantitative analysis of biofilm formation *in vitro* by CFU/mL (Log10) count for the following groups: **(A)** Mono-species biofilms formed by *C. albicans*, co-cultivated *C. albicans* plus *P. brasiliensis* biofilms or dual-species biofilms formed by the addition of *P. brasiliensis* inoculum to preformed 12 h *C. albicans* biofilms (*p < 0.001). **(B)** Mono-species biofilms formed by *P. brasiliensis*, co-cultivated *C. albicans* plus *P. brasiliensis* biofilms or dual-species biofilms formed by the addition of *P. brasiliensis* inoculum to preformed 12 h *C. albicans* biofilms. Statistical significances were calculated by using analysis of variance (ANOVA) followed by Bonferroni test.

Comparison between the *C. albicans* CFU values were similar in mono or dual-species biofilms (ANOVA followed by Bonferroni test). However, the values of dual-species biofilms when *P. brasiliensis* was added to 12 h preformed *C. albicans* biofilms were statistically significant and higher than *C. albicans* biofilm at 144 h (*p* < 0.001) (Figure 3A). Similarly, the comparison between the CFU values of *P. brasiliensis* was similar in mono or dual-species biofilms (Figure 3B). There was no significant difference in the evaluated times, suggesting that both fungi are able to co-exist in dual-species biofilms resembling those formed on oral mucosal surfaces.

### Biofilm Structure

**FIGURE 4:**
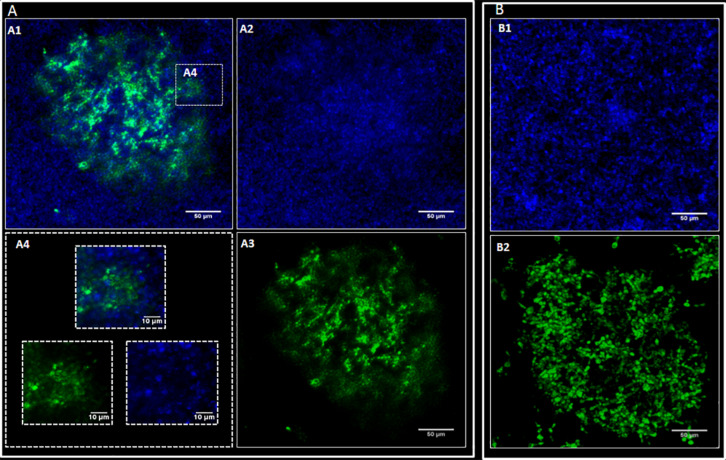
Representative confocal microscopy images. *P. brasiliensis* was marked with CFSE (green) and *C. albicans* with Calcofluor white (blue). *C. albicans* (CA) and *P. brasiliensis* (PB) mono-species biofilms formed for 48 and 144 h, respectively. For dual-species biofilms, *P. brasiliensis* previously treated with CFSE was added to 12 h preformed *C. albicans* biofilm, and incubated for 120 h. **(A)** Dual-species biofilms: overlay of CA and PB (A1), CA (A2), PB (A3) and zoom in image showing dual-species interaction (A4); **(B)** Mono-species biofilms: CA biofilm (B1) and PB biofilm (B2).

**FIGURE 5:**
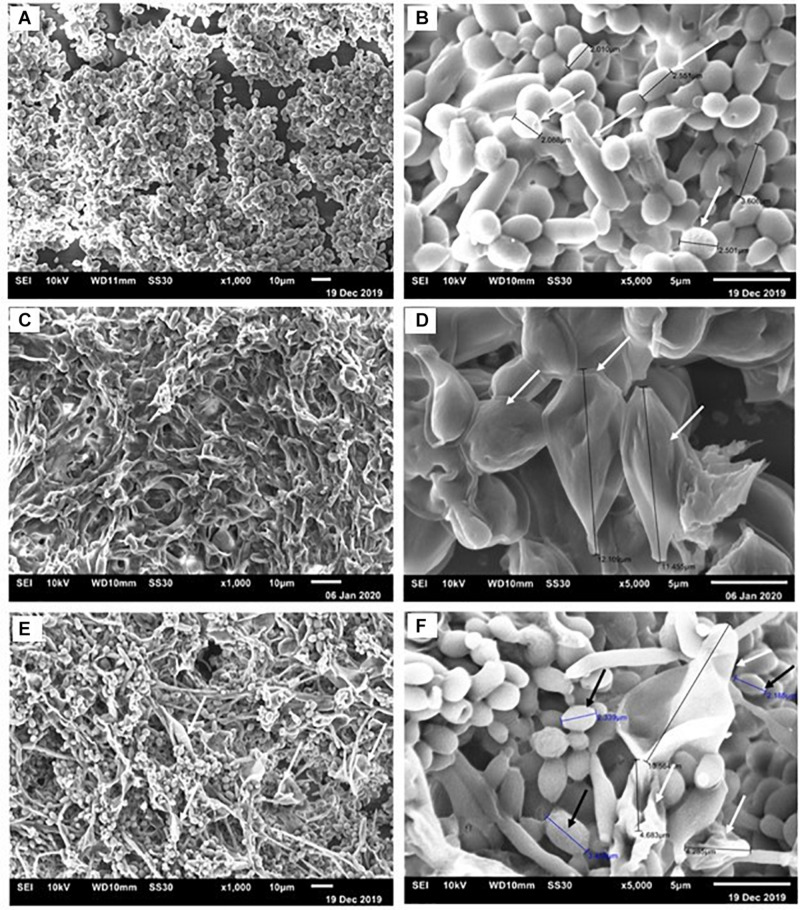
Representative scanning electron micrographs of *C. albicans* and *P. brasiliensis* mono-species biofilms formed for 48 and 144 h, respectively. For dual-species biofilms, *P. brasiliensis* was added to 12 h preformed *C. albicans* biofilm for 120 h. Biofilms formed by *C. albicans* **(A)** 1,000× and **(B)** 5,000× magnification. The white arrows indicate the yeast size **(B)**. Biofilms formed by *P. brasiliensis* **(C)** 1,000× and **(D)** 5,000×. The black arrows denote the measure of yeast **(D)**. Dual-species biofilms grown from *P. brasiliensis* added to 12 h preformed *C. albicans* biofilms **(E)** 1,000× and **(F)** 5,000×.

Confocal images showed *C. albicans* as a carpet-like biofilm (either as mono or as dual-species biofilms) whereas in dual-species biofilms, *P. brasiliensis* presented as organized clusters surrounded by *C. albicans* cells (Figure 4). The SEM images showed homogeneous and organized biofilms which were interspaced by an extracellular polysaccharide matrix (Figure 5). *C. albicans* and *P. brasiliensis* mono-species biofilms presented mainly yeasts forms (A) whilst (C) dual-species biofilms grown from *P. brasiliensis* added to preformed 12 h *C. albicans* biofilms presented a dense colonization by *C. albicans* (E). It appears that the interaction between *C. albicans* and *P. brasiliensis*, in addition to the direct physical contact, is also mediated by extracellular polysaccharides (F).

### Microbial Interaction in the *G. mellonella* Model

**FIGURE 6:**
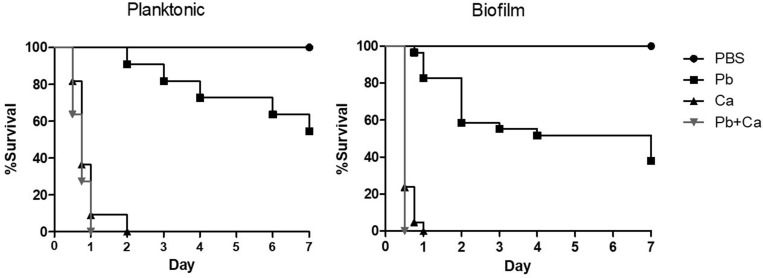
Survival curve of *Galleria mellonella* larvae infected with 1 × 10^6^ cells/larvae of planktonic cells, mono-, and dual-species biofilms grown after *P. brasiliensis* (Pb) was added to preformed *C. albicans* (Ca) biofilms. Larvae inoculated with PBS was used as control. The values are plotted as Kaplan–Meier survival curves and compared using log-rank tests.

The pathogenicity of cells released from the disrupted biofilms of *P. brasiliensis* and *C. albicans* and its co-infection were tested in the *G. mellonella* model (Figure 6). The data from larvae survival were plotted as Kaplan–Meier survival curves and compared using log-rank tests. Firstly, we observed a decrease in the survival rate of the larvae when infected with the mono- and dual-species biofilm cells compared to their planktonic cells. About 55% of the larvae were alive on the 7th day when infected with *P. brasiliensis* planktonic cells, but up to 38% were alive for its biofilm form. Likewise, infections by the two fungi in planktonic form led to the death of the larvae in the first 24 h, while larvae death was observed after 12 h of infection by the biofillm form. No larvae died with the single or double injection of PBS.

Secondly, when we compared the profile curve of larvae infected with mono- and dual-species biofilms with planktonic cells, there was a significant difference between *P. brasiliensis* and *P. brasiliensis* plus *C. albicans* (*p* < 0.05). On the other hand, the profile curve between *C. albicans* and *P. brasiliensis* plus *C. albicans* did not show a statistical difference, although the dual-species infection led to faster larvae mortality compared to *C. albicans* (1 vs. 2 days).

Thirdly, the comparison of larvae infected with mono- and dual-species biofilms cells showed a significant difference between *P. brasiliensis* or *C. albicans* and *P. brasiliensis* + *C. albicans* (*p* < 0.05).

## Discussion

*Candida* species, the most representative oral mycobiome microorganism (Bertolini and Dongari-Bagtzoglou, 2019), is normally found in the oral cavity at levels compatible to health. However, under certain conditions, especially those related to immune suppression or even in neonates or elderly, there is an increase in *Candida* carriage in the oral cavity that ultimately leads to the development of opportunistic mucosal infections (Nicolatou-Galitis et al., 2001; Bertolini and Dongari-Bagtzoglou, 2019). In this context, many studies have reported inter-kingdom *Candida*-bacterial interactions in the oral cavity, as well as *Candida-Candida* interactions and their role in oral diseases, especially at the mucosal sites (Baena-Monroy et al., 2005; Thein et al., 2009; Martins et al., 2016; Negrini et al., 2019; Pathirana et al., 2019; Rodrigues et al., 2019). Moreover, *Paracoccidioides* spp. can also be found in the oral mucosal lesions as loose or dense granulomas (de Carli et al., 2015). Thus, both fungi can be found in a suitable niche in the oral cavity to exert their pathogenicity.

Both fungi may grow as *in vitro* or *in vivo* mono-species biofilms (Hawser and Douglas, 1994; Douglas, 2003; Sardi Jde et al., 2015; Cattana et al., 2017). Therefore, evaluating the dynamics of mono- and dual-species biofilm formed by *P. brasiliensis* and *C. albicans* can widen the knowledge about the PCM infection mechanism, the most important mycotic disease in Latin America. To our knowledge, this is the first time that such dual-species biofilm has been developed and reported.

Despite the disadvantages of reproducing some biological conditions (Diaz-Garcia et al., 2019), polystyrene microtiter plates are commonly used as the standard system to study adhesion and biofilm formation by bacteria and fungi (Simoes et al., 2010; Pemmaraju et al., 2013; Sardi Jde et al., 2015).

Both biomass and biofilm metabolic activity were used in this study to characterize the dynamics of biofilm growth. Based on the quantification of biomass, we found that *C. albicans* mono-species biofilms reached a mature stage from 72 to 96 h in agreement with previous data (Pierce et al., 2010; Cavalheiro and Teixeira, 2018). *P. brasiliensis* needed 144 h to reach its highest biomass, which corroborates with the previous studies carried out by Sardi Jde et al. (2015). In addition, the *C. albicans* mono-species biofilm showed higher values of biomass, metabolic activity and CFU than that found in the *P. brasiliensis* mono-species biofilm in most of the evaluated times.

Interestingly, the growth dynamics of dual-species biofilms was different. The mature stage (in terms of biomass) of co-cultivated dual-species biofilms was reached after 96 h of growth, while the dual-species biofilm formed after *P. brasiliensis* was added to 12 h preformed *C. albicans* biofilm reached its highest biomass in 48 h. In line with the fast growth of dual-species biofilms, their metabolic activity was also higher than the mono-species biofilms, almost at all time points, mainly in 72 h. In agreement with the biomass and metabolic activity results, the *C. albicans* CFU values in dual-species biofilms were higher compared to the CFU values of mono-species biofilms, specially from 96 h. The increase in metabolic activity, biomass and CFU, primarily in the dual-species biofilms grown after *P. brasiliensis* was added to preformed 12 h *C. albicans* biofilms, compared to *P. brasiliensis* mono-species biofilms, indicate that there were no loss to *P. brasiliensis* and that can coexist in the same environment as *C. albicans*.

There are common functional organization principles that occur at sites of polymicrobial infection, including exploration of metabolites, immunological modulation, niche optimization and induction of virulence. Thus, the presence of a microorganism can generate a niche for other pathogenic microorganisms or predispose the host to colonization by other microorganisms, or two or more non-pathogenic microorganisms together can cause disease (Brogden et al., 2005; Peters et al., 2012). Clinically, we could hypothesize that oral cavity lesions caused by *P. brasiliensis* would be more aggressive when both fungi (*C. albicans* and *P. brasiliensis*) are present at both the same time and site, once we observe an improvement in the biofilm features. The surface first settled by *C. albicans* cells has already been related to increased adhesion, accompanied by a significant increase in cell viability with bacterial biofilms (Bartnicka et al., 2019). Interactions in mixed biofilms are essential to produce a rich exopolysaccharide matrix. In a previous study, the synergism between *C. albicans* and *Streptococcus mutans* increased the virulence of mixed biofilms formed on the surfaces of teeth, thus contributing to the severity of caries (Falsetta et al., 2014; Sztajer et al., 2014). Here, the enhanced growth through intergeneric cooperation may be taking place and the damage to oral mucosa tissues caused by *Candida* may be influence the PCM pathogenesis.

The SEM images of mono-species biofilms showed *C. albicans* and *P. brasiliensis* yeasts embedded in extracellular polysaccharide matrix and presenting an average size from 2 to 4 μm and from 11 to 12 μm, respectively. Dual-species biofilms also showed similar features of mono species biofilms, however, presenting a higher proportion of *C. albicans* yeast cells. This higher proportion of *C. albicans* may be due to the slower growth of *P. brasiliensis*. Interestingly, the interaction between *C. albicans* and *P. brasilensis* in dual-species biofilms seem to happen both by direct physical contact and mediated by the extracellular polysaccharide matrix. It is known that some *Candida* surface adhesins, such as Als3, exert an important role on *Candida* biofilm formation as well as mediating the co-aggregation between *Candida* and other microorganisms (Verstrepen and Klis, 2006; Cavalheiro and Teixeira, 2018; Negrini et al., 2019). Moreover, *Candida* cell-wall mannan-residues might also act as an adhesion element for extracellular polysaccharides (Negrini et al., 2019). In addition, several *Paracoccidioides* adhesin proteins have been shown to be extracellular matrix ligands, such as 43 kDa glycoprotein (gp43), enolase and glyceraldehyde-3-phosphate dehydrogenase (GAPDH) (Vicentini et al., 1994; Mendes-Giannini et al., 2004; Barbosa et al., 2006; Donofrio et al., 2009). These adhesins may be used in the adhesion process, the initial phase of biofilm formation. In recent studies, GP43, GAPDH, and proteinase genes were considered to be overexpressed in the biofilm (Sardi Jde et al., 2015). Then, we hypothesize that agglutinin-like sequence (Als) surface proteins optimize the physical interaction and co-aggregation between *C. albicans* and *P. brasiliensis* and the cell-wall mannan-residues might act as a receptor to extracellular polysaccharides, which in turn act as a bridge, facilitating the adhesion between both microorganisms. It is unclear though, at this moment, which of those adhesion mechanisms are the most predominant and whether other adhesion mechanisms might be involved in such interactions. These interactions between two organisms can induce additional responses among themselves and modify the immediate environment to influence pathogenesis. Although we have begun to investigate the consequences of these interactions, much is still unknown. This highlights the need for future mechanistic studies to be properly designed to address those questions.

*Galleria mellonella* has been presented as a useful invertebrate model to assess the fungi virulence, innate immune response and antifungal efficacy due to the ease of breeding it in the laboratory as well as in carrying out experiments with the larvae (Fuchs and Mylonakis, 2006). The model is especially advantageous in the study of dimorphic fungi due to the possibility that the larvae are kept at 37°C during survival assays, which represents the conditions of human physiology and contributes to maintaining the yeast phase of fungi such as *Paracoccidioides* spp. (Singulani et al., 2018). More recently, *G. mellonella* has been used to evaluate co-infection between fungi (Alcazar-Fuoli et al., 2015) or fungus and bacteria (Kean et al., 2017; Rossoni et al., 2017; Reece et al., 2018; Sheehan et al., 2020). Furthermore, some results are similar to those found in mammal models (Sheehan et al., 2020). Here, we used the *G. mellonella* model to evaluate the virulence of mono-species and dual-species *P. brasiliensis* and *C. albicans* biofilm formation. Cells from biofilms usually exhibit different phenotypes compared to planktonic cells and are associated with a higher pathogenicity of the microorganisms, including resistance to host immune mechanisms (Martinez and Fries, 2010). In this aspect, our results showed that cells released from the disrupted biofilms increased the virulence of both fungi compared to their planktonic cells and consequently, it decreased the survival rate of the larvae. Similar results were presented for *Cryptococcus neorfomans* in a previous study (Benaducci et al., 2016). Furthermore, the comparison of larvae infected with mono-species and dual-species biofilms presented the difference between *P. brasiliensis* or *C. albicans* and *P. brasiliensis* plus *C. albicans*, especially for biofilm formation. This means that the larvae survival on dual-species biofilms was lower when compared to survival with mono-species biofilms. Thus, our results from *G. mellonella* are based on *in vitro* assays since dual-species biofilms showed greater biomass and metabolic activity, and therefore were more virulent than mono-species biofilms. Finally, our data may indicate that *P. brasiliensis* and *C. albicans* co-existence is likely to occur on oral mucosal as biofilms, using an *in vivo* model. In the same way, when G. *mellonella* larvae were infected with *C. albicans* and *Staphylococcus aureus*, there was an increase in the virulence of these microorganisms and the antifungal resistance, which indicated a synergic interaction (Kean et al., 2017).

One may argue that the dynamics of dual-species biofilm growth could be affected by the different growth kinetics of *P. brasiliensis* and *C. albicans* and that both strains should ideally present similar growth curves. We acknowledge that the maturity of *P. brasiliensis* mono-species biofilms was reached later compared with *C. albicans* biofilms. This way, we might consider the contribution of *C. albicans* to dual-species biofilms (in terms of biofilm dry weight, biofilm metabolic activity and infectivity on the *G. mellonella* model) to be greater than *P. brasiliensis*. *Paracoccidioides* and *Candida* are often co-isolated in the oral mucosa. This presence may represent a polymicrobial association with mutual collaboration or advantage for one of the agents, that can lead to more exacerbated clinical and pathological manifestations. Moreover, the comparison of larvae infected with mono- and dual-species biofilms cells showed a significant difference between *C. albicans* and *P. brasiliensis* plus *C. albicans* (*p* < 0.05), which can be a consequence of virulence factors and *quorum sensing* mechanisms of biofilms.

Irrespective of any difference in growth kinetics, both the strains were able to co-exist on the studied dual-species biofilms, suggesting that a fungal-fungal interaction is likely to occur on oral mucosal sites. In fact, an *in vivo* rodent model should be adopted in further studies to assess both the infectivity of this dual-species biofilms on oral mucosal surfaces and the elicited immune-response against those fungal biofilms. Thus, a deeper understanding of the adhesion and signaling mechanisms involved in the interactions between both fungi will provide a new perspective on the role of known virulence determinants and the relevant factors for both diseases.

## Conclusion

This study suggested that the *C. albicans* and *P. brasiliensis* can coexist in the same environment and that a fungal-fungal interaction is likely to occur on oral mucosal sites. These data widen the knowledge associated with the dynamics of fungal biofilm growth that can potentially lead to the discovery of new therapeutic strategies for fungal infections.

## Data Availability Statement

All datasets generated for this study are included in the article/supplementary material.

## Author Contributions

LO and MMG contributed to the research idea and experimental design. LO, KM-A, and NF performed the experiments. LO, KM-A, JS, NF, and RA analyzed the data, prepared, and wrote the manuscript. RA, AF-A, RP, and MMG revised the final draft of the manuscript. All authors contributed to the article and approved the submitted version.

## Conflict of Interest

The authors declare that the research was conducted in the absence of any commercial or financial relationships that could be construed as a potential conflict of interest.
